# Supplementary material for: LFA-1: A potential key player in microglia-mediated neuroprotection against oxygen-glucose deprivation in vitro
Source: PLoS One. 2025 Jan 9;20(1):e0314020. doi: 10.1371/journal.pone.0314020 (PMC11717251; doi:10.1371/journal.pone.0314020)
Supplement: Attachment — Submitted filename: Response to Reviewers_rebuttalletter.docx [file pone.0314020.s001.docx]

# **Response to Reviewers**

**Dear academic editors, dear reviewers,**

Please find attached the updated manuscript and a version in track change mode with the changes highlighted in yellow as discussed. You will also find the answers to the reviewers' questions below. All answers are included in the uploaded manuscript files.

**Reviewer #1:** This study shows the critical role of the β2 integrin, LFA-1, in microglia-mediated neuroprotection during oxygen-glucose deprivation (OGD) in hippocampal cell cultures. The research demonstrates that LFA-1 is necessary for neuronal survival under ischemic conditions, as its inhibition with the compound BIRT377 resulted in a dose-dependent increase in neuronal death. ICAM-5, which is strongly expressed in hippocampal neurons, was identified as a potential ligand for LFA-1, indicating an important interaction between microglia and neurons that is essential for neuroprotection. These findings highlight the significance of LFA-1 in neuroprotective signaling and emphasize the need for further investigation into integrin-mediated pathways in the context of ischemic stroke.

**Minor concerns:**

In the introduction, it is mentioned that IGF-1 is an example of a neurotransmitter, but its canonical function is as a growth factor mainly released from the liver, although also produced in many tissues. IGF-1's main activity is to induce proliferation; however, in the brain, it has been related to survival and neuroprotection.

***What happens to the BV-2 microglial cells that are co-incubated with the hippocampal organotypic cultures? Is there any kind of tissue penetration?***

**Answer:** Using two-photon microscopy, we demonstrated that added BV-2 microglia penetrates the slice in a time-dependent manner following a OGD [1]

***Do microglial BV-2 cells secrete proinflammatory cytokines? Are they M1 or M2 phenotype?***

**Answer:** Measuring cytokines was not within the scope of this study; therefore, we did not include it. While we have not determined the microglia phenotype, we assume that they are initially M1 polarized.

***Are they pre-treated to express a specific phenotype?***

**Answer:** No, the BV-2 microglia were not pre-treated.

***Did you try higher doses of BIRT377?***

**Answer:** No, we did not apply higher doses.

***Is PI incorporation specific to neuronal cells?***

**Answer:** No, PI intercalates into the DNA of every necrotic cell.

***Please include better pictures of the OHCs in Figures 2C and 3C, as they are very small, and without nuclei counterstaining, it is very hard to identify the damage zone and the affected cells.***

**Answer**: PI is a nuclear counterstain.

***Authors show that N=7. How was this calculated? What is the effect size? Was it calculated a priori?***

**Answer:** We need to apologize. The number of slices were false. We corrected the numbers (higher) in the manuscript.

***Does BIRT377 have a differential effect on endogenous microglia and BV-2?***

**Answer:** We did not differentiate between the two types. However, the data indicate that BIRT377 had an effect in both conditions: ODG with endogenous microglia (Figure 2) and exogenous microglia (Figure 3). This suggests that BIRT377 acts similarly on both types of microglia.

**Major concerns:**

The abstract is very general and does not provide important information such as methodology, a summary of results, and a brief conclusion. It also states that there is no single therapy to induce neuroprotection after a stroke in patients, which is hard to believe. I recommend being more cautious with statements like this.
The control in Figures 2C and 3D show a strong discrepancy in propidium iodide incorporation intensity. The timeline indicates that they are exposed to PI for 24 hours; if there is any other difference in the protocol that explains this fluorescence inconsistency, it should be included in the manuscript.

***I strongly recommend including a conclusion or summary at the end of the discussion, which includes only technical English, avoiding expressions such as “Like a stone into water.” This can be confusing for some international readers.***

**Answer***: We adapted the manuscript accordingly.*

***Stressed microglia are reported to promote an M1 proinflammatory phenotype, which can be harmful to neural tissue. Why does BIRT377, once blocking OGD in non-stressed BV2, increase survival (Figure 2B)? Also, endogenous microglia and BV2 are likely to have opposing responses since one is stressed and the other is not. Please include a discussion about this.***

**Answer:** BIRT377 blocks LFA-1(CD11a) which is crucial for migration of externally added microglia into the slice. [1] Our ex vivo studies demonstrated that the migration of exogenous microglia into the slice is essential for exerting neuroprotection [1,2]. Additionally, other studies have shown that the ablation of endogenous microglia in vivo exacerbates neuronal damage following experimental stroke. Both observations indicate that both endogenous and exogenous microglia are neuroprotective rather than destructive in stroke models [3]Both observations indicate that endogenous, as well as exogenous microglia are neuroprotective rather than destructive in stroke models. These aspects are included in the discussion.

***The number of results seems a little short. It would be interesting to see what the mediators are in the effect exogenous microglia upon survival in hippocampal tissue cultures. Maybe qPCR or ELISA for cytokines and neurotrophins could complement this work.***

**Answer:** We regret that the project has been terminated. Future studies will aim to delve deeper into the underlying mechanisms; we hope we can conduct such study in short term.

**Reviewer #2:** In this manuscript, the authors investigated LFA-1 inhibition with BIRT377 on neuronal death after oxygen-glucose deprivation (OGD) in cultured hippocampal slices. The authors found that BIRT377 treatment enhanced neuronal death after OGD, and abolished the neuroprotective effect of transplanted BV2 cells on OGD-preconditioned slices. Below are some comments on the manuscript.

***(1) In figure 1C, the authors show expression of ICAM5 but not ICAM1 in neurons. At which experimental conditions (OGD vs. Normal) was this done?***

**Answer:** It was done under normal conditions.

***(2) In figure 3, the authors show that BV2 cell transplantation is neuroprotective on OGD-preconditioned slices, which can be partially abolished by LFA-1 inhibition. The expression of LFA-1 in BV2 cells should be experimentally confirmed.***

**Answer:** The expression of LFA-1 has been documented in other publications [4,5]. We conducted CD11a (LFA-1) antisense knockdown in BV-2 microglia, which resulted in impaired microglial migration [1]. This information has been included in the manuscript.

***(3) There is a lack of scale bars on the immunofluorescent images in all figures.***

**Answer:** We added the scale bars.

***(4) It is difficult to see the morphology of the slices in figure 2C and figure 3C. The authors should either provide bright-light images of the slices together with the fluorescent images or enhance the intensity of the fluorescent images to show the slice morphology.***

**Answer:** We enhanced the quality of images.

***(5) The manuscript needs careful proof-reading: in several occasions, the authors start the sentence with “previously”, “previous studies” or “previous data” without providing any literature reference; in line 7 of the abstract: “hippocampal cell culture” should be “hippocampal slice culture”; in line 16 of the introduction, IGF-1 is not a neurotransmitter; in the “Organotypic hippocampal slice cultures” section of the method: repeated texts in lines 5-8; for microglia labelling: it is stated in the main text that rhodamine-IB4 was used; however, in the method section it is indicated that Alexa568-IB4 was used.***

**Answer:** We completed proofreading and made changes according to your addressed points.

## **References:**

1.	Neumann J, Gunzer M, Gutzeit HO, Ullrich O, Reymann KG, Dinkel K. Microglia provide neuroprotection after ischemia. FASEB Journal. 2006;20. doi:10.1096/fj.05-4882fje

2.	Mitrasinovic OM. Microglia Overexpressing the Macrophage Colony-Stimulating Factor Receptor Are Neuroprotective in a Microglial-Hippocampal Organotypic Coculture System. Journal of Neuroscience. 2005;25: 4442–4451. doi:10.1523/JNEUROSCI.0514-05.2005

3.	Lalancette-Hebert M, Gowing G, Simard A, Weng YC, Kriz J. Selective Ablation of Proliferating Microglial Cells Exacerbates Ischemic Injury in the Brain. Journal of Neuroscience. 2007;27: 2596–2605. doi:10.1523/JNEUROSCI.5360-06.2007

4.	Paetau S, Rolova T, Ning L, Gahmberg CG. Neuronal ICAM-5 inhibits microglia adhesion and phagocytosis and promotes an anti-inflammatory response in LPS stimulated microglia. Front Mol Neurosci. 2017;10. doi:10.3389/fnmol.2017.00431

5.	Hailer NP, J T JI, Nitsch R. Resting Microglial Cells In Vitro: Analysis of Morphology and Adhesion Molecule Expression in Organotypic Hippocampal Slice Cultures. Glia. 1996.
